# Contemporary Magnetic Removable Partial Denture Utilizing a Novel Ultra-Thin Magnetic Attachment System

**DOI:** 10.3390/dj13070278

**Published:** 2025-06-20

**Authors:** Adityakrisna Yoshi Putra Wigianto, Yuichi Ishida, Kohei Kamoi, Takaharu Goto, Kazumitsu Sekine, Megumi Watanabe, Tetsuo Ichikawa

**Affiliations:** 1 Department of Prosthodontics & Oral Rehabilitation, Tokushima University Graduate School of Biomedical Sciences, Tokushima 770-8504, Japan; adityakrisnayoshi@tokushima-u.ac.jp (A.Y.P.W.); junchan@tokushima-u.ac.jp (Y.I.); tak510@tokushima-u.ac.jp (T.G.); megwat@tokushima-u.ac.jp (M.W.); 2 Dental Technology Section, Division of Clinical Technology, Tokushima University Hospital, Tokushima 770-8504, Japan; dt-k.kamoi@tokushima-u.ac.jp; 3 Department of Biomaterials and Bioengineering, Tokushima University Graduate School of Biomedical Sciences, Tokushima 770-8504, Japan; ksekine@tokushima-u.ac.jp

**Keywords:** magnetic attachment, telescopic crown, denture retention, removable partial denture, CAD/CAM

## Abstract

**Background/Objectives:** Recently, a novel magnetic attachment system was introduced to improve performance. Using the same technology, a new ultra-thin magnetic attachment (UTMA) was possible to produce. This study aimed to evaluate the feasibility of a magnet-retained telescopic partial denture (MTPD) utilizing the new UTMA. **Methods**: This in vitro study was performed using a titanium master model representing prepared lower first-premolar and second-molar abutment teeth. The inner crowns (ICs) (h: 4 mm, 4° taper) and four-unit MTPDs were fabricated via computer-aided design/computer-aided manufacturing (CAD/CAM) using zirconia. A Ø4 mm UTMA system (magnet assembly and keeper thickness: 0.6 mm and 0.4 mm, respectively) was cemented into the MTPD and the ICs using dual-cure resin cement. A load of 100 N was applied along with 10,000 insertion–removal cycles. The MTPD retentive force was measured before and after every set of 1000 cycles. Stability tests and surface morphology evaluations were conducted before and after cycling. A paired *t*-test (α = 0.05) was used to observe statistical differences. **Results**: The average retentive force of the MTPD was 6.86 ± 0.63 N and did not change significantly (*p* > 0.05) following the load cycles (6.66 ± 0.79 N). The MTPD demonstrated adequate stability under the occlusal load. Minimal deformations were observed on the magnet assemblies, keepers, ICs, and MTPD surfaces after the load tests. **Conclusions**: Considering the limitations of this study, an MTPD utilizing novel UTMAs fabricated through a digital workflow demonstrated adequate retentive force, stability, and durability for clinical use.

## 1. Introduction

Magnetic attachment has garnered attention due to the development of neodymium ferrite boron (NdFeB) magnets and laser welding, which provide a lasting magnetic force without corrosion. Magnetic attachments generally provide a retentive force by utilizing the attractive force between two components: a magnetic assembly (MA) embedded inside the denture base and a keeper (KP) cemented to the abutment tooth [1,2]. Moreover, magnetic attachments offer advantages such as minimum lateral and rotational stress to the abutment tooth, constant and durable retentive force, excellent esthetics, and easy maintenance [3]. Nevertheless, the placement of the MA and KP also sacrifices denture or artificial tooth thickness, which makes that area prone to denture fracture, as reported in a previous study [4]. Makihara et al. [5] reported the modification of a telescopic crown equipped with magnetic attachments, which resulted in satisfactory results for both patients and dentists. Unlike a conventional telescopic crown’s retentive force that might decrease owing to wear of the frictional walls, a magnetic telescopic crown denture might offer advantages in terms of a stable retentive force and easier adjustment; however, such crowns require spacious occlusal clearance on the abutment tooth.

Currently available magnetic attachment systems have an MA thickness of 1 mm or more [6,7,8,9]. The most recent developments in magnetic attachment structures using novel manufacturing techniques have been reported. This innovation eliminated the necessity for a separate nonmagnetic disc component in the MA, reducing the number of components from four to three; thus, a more compact MA structure was achieved. Consequently, reduced fabrication costs can be achieved with the aforementioned system while providing favorable retentive force and durability [9]. By leveraging the same technology (high-frequency heat treatment, stamp manufacturing, stainless-steel magnets), along with a custom ring-shaped thin NdFeB magnet invention, a prototype of an ultra-thin magnetic attachment (UTMA) system has been developed. This system features a thickness of 600 µm for the MA and 400 µm for the KP. The main clinical advantage of a UTMA lies in its ultra-thin profile, which requires significantly less vertical clearance compared to conventional attachments. This minimum low-clearance-space requirement enables increased preservation of the abutment teeth and prosthesis thicknesses, which is especially beneficial in cases with limited occlusal clearance where installing an attachment has been impossible in the past. Consequently, this advancement increases the potential for broader applications of the UTMA system across various clinical situations, especially for patients with a reduced vertical dimension, or in cases where ease of repair, esthetic appearance, and thorough prosthesis hygiene control are prioritized.

In this study, the UTMA system is utilized in a telescopic partial denture without a denture base, namely the so-called magnet-fixed telescopic partial denture (MTPD). However, the thin structure of this magnet could pose challenges in handling during prosthesis fabrication and in terms of surface integrity and retentive-force durability. Therefore, further development of the prosthesis fabrication process is required. In addition, an in vitro evaluation of the performance and durability of a prosthesis is essential before proceeding to clinical trials.

Regarding the fabrication process, the goal of achieving a maximum retentive force for a denture with a magnetic attachment necessitates precision and technical sensitivity. This raises the concern that conventional fabrication methods such as the lost-wax technique could introduce the risk of technical errors, potentially hindering the attainment of an optimal retentive force. Over the last few decades, computer-aided design and computer-aided manufacturing (CAD/CAM) technology has been widely employed in dentistry because it can simplify techniques, minimize laboratory errors, reduce clinical time, and improve the accuracy and fit [10,11]. Therefore, the advantages of CAD/CAM technology might help in the fabrication of a dental prosthesis with a UTMA, with more precise design planning and predictable results.

This study aimed to develop a UTMA-retained MTPD with a digital workflow and evaluate the feasibility and durability in vitro. The primary outcome was retentive force, and the secondary outcome was prosthesis stability, assessments of which were carried out before and after the durability test by applying load and insertion–removal cycles. The null hypothesis was that there is no retentive-force difference with the MTPD utilizing a UTMA before and after the insertion–removal cycles.

## 2. Materials and Methods

### 2.1. Magnetic Attachment

**Figure 1:**
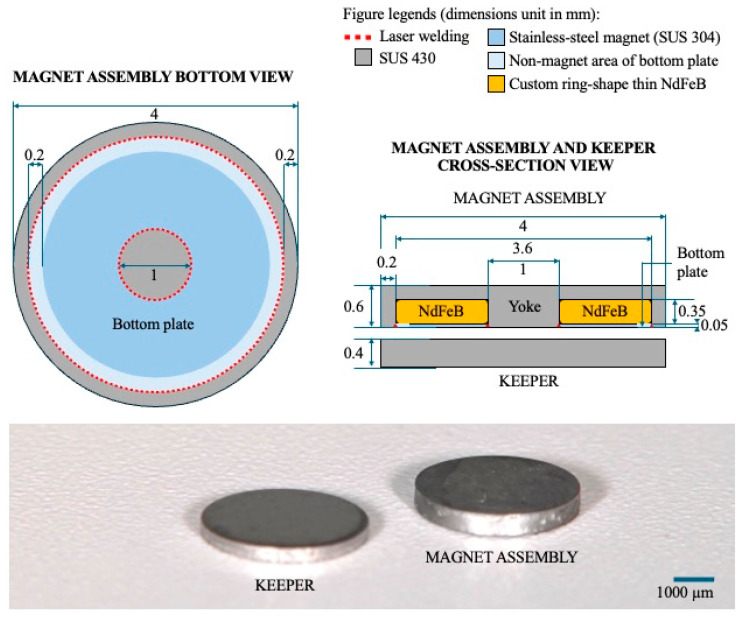
Illustration of the UTMA’s structure and dimensions in mm, and a photograph of the MA and KP.

The UTMA system (Magteeth MTs600, Magnedesign Co., Aichi, Japan) is illustrated in Figure 1.

### 2.2. Fabrication of a UTMA-Retained MTPD

**Figure 2:**
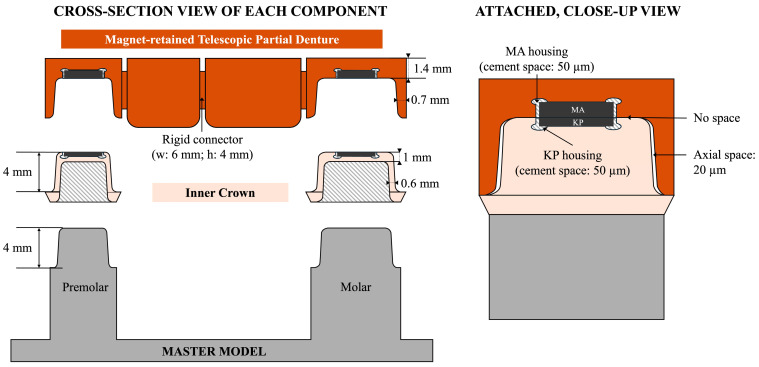
Illustration of the master model, IC, and MTPD dimensions and design evaluated in this study (left: unattached, cross-section view; right: attached, close up view).

A total of five MTPD specimens utilizing ten UTMAs were prepared and tested in this study. This sample size was determined based on precedents in similar previous in vitro mechanical evaluations of dental attachments and telescopic/double crowns. The experimental MTPD comprised two abutment outer crowns (OCs) positioned on the first premolar and second molar, and two pontic crowns on the second premolar and first molar, as displayed in Figure 2. The KP and MA were placed on the inner crowns (ICs) and OCs, respectively, with the crowns being crafted from zirconia. The master model was designed using Autodesk Fusion 360 (Autodesk, San Francisco, CA, USA) to represent prepared abutments of first-premolar (dimension at cervix = mesiodistal: 6.5 mm; buccolingual: 7.5 mm) and a second-molar morphology (mesiodistal: 9 mm; buccolingual: 8 mm). Thereafter, fabrication was performed using titanium discs (KZR-CAD Ti, Yamakin, Kochi, Japan) via computer numerical control (CNC) milling. A laboratory scanner (E4, 3Shape, Copenhagen, Denmark) was used to obtain the standard tessellation language file of the master model for prosthesis design.

The ICs and MTPD were custom-designed using general-purpose CAD software with a touch haptic device (Geomagic Freeform version 2022.1.32, 3D Systems, Rock Hill, SC, USA) and dental CAD software (3Shape Dental System, 3Shape, Copenhagen, Denmark). The wall height of each IC was 4 mm with a 4° taper and deep chamfer margin shape, with a 0.6 mm thickness. In the center of the IC occlusal surface, a housing for fixating a KP with a 50 µm cement space was prepared (Figure 2).

**Figure 3:**
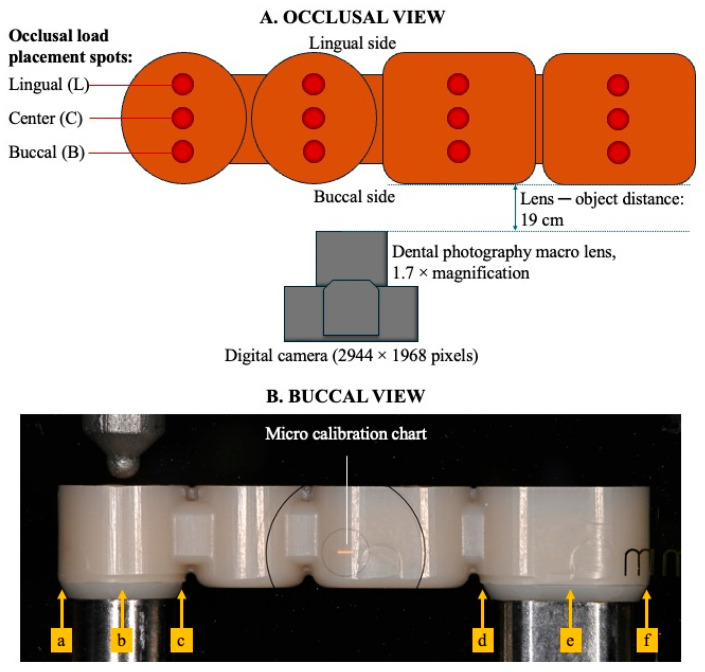
(**A**) Occlusal view of the MTPD surface with stoppers (∅1.5 mm; depth: 0.2 mm) for placing the load on 12 spots; (**B**) buccal view of photograph of the sample installed in the universal testing machine for stability analysis. Yellow arrows represent areas of interest for margin movement evaluation. a: mesial part of premolar; b: center part of premolar, c: distal part of premolar; d: mesial part of molar; e: center part of molar; and f: distal part of molar IC-OC interface.

The intaglio surface design of the MTPD with a 0.7 mm minimum thickness was initially fabricated with dental CAD software with a 20 µm space (no frictional contact) to the IC. Furthermore, to prevent the formation of a vertical gap between the MA and KP during the fixation process, the occlusal space between the IC and MTPD was eliminated by incorporating a custom housing design with a 50 µm cement space for fixating the MA using general-purpose CAD software. In this study, the simple outer morphology of the MTPD was designed for multiple evaluation purposes. The MTPD occlusal surface was flat to facilitate its fixation to the upper jig for retentive-force measurement and insertion–removal cycles. In addition, round stoppers with a 1.5 mm diameter and 0.2 mm depth were made to guide load placements for the MTPD stability evaluation (Figure 3).

The ICs and MTPDs were fabricated using 4 mol% yttria-partially stabilized zirconia (4Y-PSZ) discs (Katana zirconia HT, Kuraray Noritake Dental, Tokyo, Japan) via CNC milling (MD-500, Canon, Tokyo, Japan). Afterward, the sintering was performed according to the manufacturer’s instructions using a sintering furnace (F-1N, Kuraray Noritake Dental, Tokyo, Japan). The process involved heating up to 1500 °C, with a dwelling time of 1 h 30 min, and a cooling rate of 5.56 °C/min. Ten ICs and five MTPDs were fabricated by a dental technician.

The fitness of the ICs and MTPDs was thoroughly evaluated to the master model. The intaglio surface of the ICs and the abutment teeth underwent sandblasting with 50 µm aluminum oxide at 0.3 MPa, then was cleaned with alcohol in an ultrasonic cleaner. A universal prosthetic primer containing 10-methacryloyloxydecyl dihydrogen phosphate (MDP) (Clearfil ceramic primer plus, Kuraray Noritake Dental, Tokyo, Japan) was applied to the sandblasted surface, followed by IC cementation to the master model using a dual-cure resin cement (Panavia V5, Kuraray Noritake Dental, Tokyo, Japan).

Try-in of the MA and KP into the ICs and MTPD was performed to ensure a good fit in the housing before fixation with cement. This step also verified the marginal fit between the MTPD and ICs when the MAs and KPs were positioned. The MA, KP, and housing surfaces were sandblasted using the aforementioned procedure. Following primer application, the KP was cemented using the following procedure to achieve a flat surface with the ICs: a microscope glass cover was placed between the MA and KP, and cementing of the KP, light-curing for 10 s (Pencure II, Morita, Tokyo, Japan), and removing the excess cement were carried out. Finally, the MAs were cemented into the MTPD by placing them on the KP. Then, an adequate amount of cement was placed in the MTPD housing. The MTPD was carefully positioned on the ICs and held steady for more than 3 min. The magnetic attachment fixation procedure was performed based on a method recommended by the Japanese Society of Magnetic Applications in Dentistry [12] with some modifications.

### 2.3. Evaluations

#### 2.3.1. Accuracy

To assess the accuracy of the design and fabrication protocol, the margin gap between the IC and MTPD was evaluated before and after KP and MA fixation, using a digital stereomicroscope (VHX-F, Keyence, Osaka, Japan).

#### 2.3.2. Retentive Force of the UTMA

Before fixing the MA to the MTPD components, the performance of each sample was evaluated by measuring its retentive force and magnetic field strength. The MA adhered to an acrylic rod using a cyanoacrylate adhesive (Aron alpha, Toagosei, Tokyo, Japan) installed in the tensile attachment on the upper jig of a precision universal testing machine (AG-X 1kN, Shimadzu, Kyoto, Japan). The KP was fixed to a flat specimen table on the lower jig using the same method. The retentive force was measured according to ISO 13017:2020 [13] using the tensile mode with a crosshead speed of 2 mm/min. The maximum force required to detach the MA from the KP was recorded as the retentive force. A Gaussmeter equipped with an axial probe (GM-310, Denshijiki Co. Ltd., Tokyo, Japan) was utilized to measure the magnetic field strength in milli Tesla (mT).

#### 2.3.3. Retentive Force and Stability of the MTPD

The samples were fixed to the bottom jig of a precision universal testing machine using fixation screws. The acrylic block was fixed to a tensile-evaluation attachment connected to a load cell. The MTPD was fixed to an acrylic block using cyanoacrylate adhesive. The crosshead speed was set to 2 mm/min, as described in ISO 13017:2020 [13]. The maximum force required to detach the MTPD from the ICs was recorded as the retentive force. Five measurements were performed for each sample.

Using a precision universal testing machine, a 100 N load with a crosshead speed of 15 mm/min was applied vertically to the MTPD occlusal surface on 12 prepared spots (Figure 3A). A 0.01 mm calibration chart (MJ-MS27, Sato Tech, Tokyo, Japan) was used to calibrate the margin gap. The prosthetic movements were measured using two-dimensional images of the MTPD margin movement under load using a digital camera (Nikon Z-5, Nikon, Tokyo, Japan) equipped with a dental photography lens (DCN-GP/DUW, Sonic Techno, Tokyo, Japan) with 1.7× magnification. The images were acquired at three timestamps: before, during, and after loading. Thereafter, the margin gaps from the buccal side were measured using image analysis software (ImageJ version 1.54, NIH, Bethesda, MD, USA) on the area of interest, as shown in Figure 3B. Analysis was performed before and after the durability tests.

#### 2.3.4. Durability Test

Using the cyclic mode of the precision universal testing machine, a durability test was performed by applying insertion and removal cycles with a 100 N load at a crosshead speed of 15 mm/min for a total of 10,000 cycles, in line with the procedure described in our previous study [9]. After every 1000 cycles, the retentive force of the MTPD was measured. The surface conditions of the KP, MA, IC, MTPD, and MTPD–IC interfaces were evaluated using a digital stereomicroscope at baseline and after 10,000 cycles.

### 2.4. Statistical Analysis

SPSS ver. 25 (IBM, Armonk, NY, USA) was used for the statistical analysis. The normality of the differences between insertion–removal cycles was confirmed via Shapiro–Wilk tests (*p* > 0.05 for all comparisons). To compare the MTPD mean retentive force across cycles, a paired *t*-test was performed with a 95% confidence interval. This statistical test was used to analyze the collected datasets from various cycles applied, allowing for a comparison of changes in the retentive force over time, with significance set at α = 0.05.

## 3. Results

**Figure 4:**
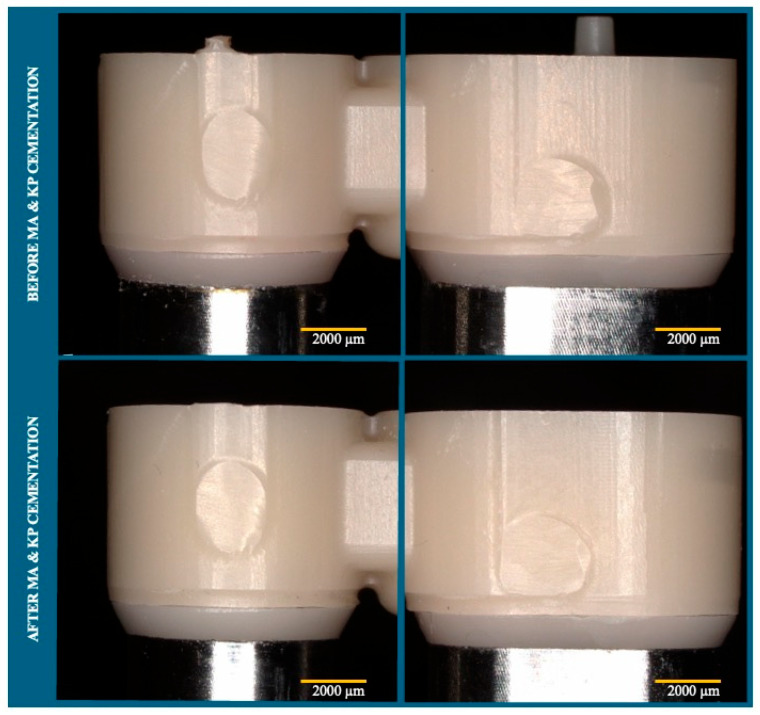
Representative images of the margin evaluation of the MTPD taken by digital stereomicroscope before and after MA and KP cementation (left: first premolar: right: second molar).

MTPD fabrication using CAD/CAM with a modified housing design for the UTMA resulted in predictable and accurate results, as indicated by the consistent fit between the abutment tooth–IC and IC–MTPD across all specimens. The proposed housing design also aided MA and KP placements with minimum adjustments. No change in the MTPD–IC marginal fit before and after MA and KP fixation was observed, as displayed in the microscopic images in Figure 4.

**Figure 5:**
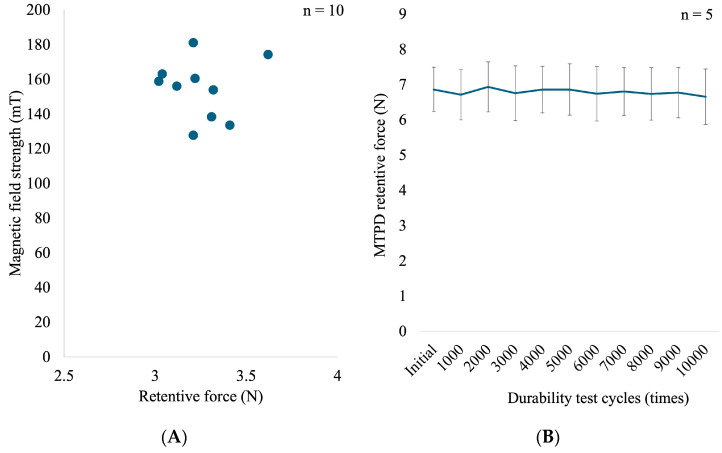
(**A**) Scatter plot showing the relationship between the magnetic field strength and the corresponding retentive force of UTMA samples. (**B**) Line diagram representing MTPD retentive force means and standard deviations from the baseline through the number of durability test cycles applied.

Table 1 summarizes the magnetic-field strength and retentive forces of each UTMA used in the MTPD. The UTMA’s mean magnetic field strength was 154.72 ± 17.13 mT, and the retentive force was 3.25 ± 0.18 N. The value of the magnetic field strength varied widely but did not align with the retentive-force value (samples with a high magnetic field strength did not exhibit a high retentive force and vice versa). Figure 5A provides the visualization of the UTMA retentive force in relation to the magnetic field strength, and Figure 5B demonstrates the mean retentive force of the MTPD at baseline and after every 1000 cycles. The data were normally distributed (*p* > 0.05). The initial retentive force was 6.86 ± 0.63 N and did not change significantly (*p* > 0.05) throughout the durability test until 10,000 cycles (6.66 ± 0.79 N). The effect size, calculated using Hedge’s g, was 0.35, indicating a small effect.

**Figure 6:**
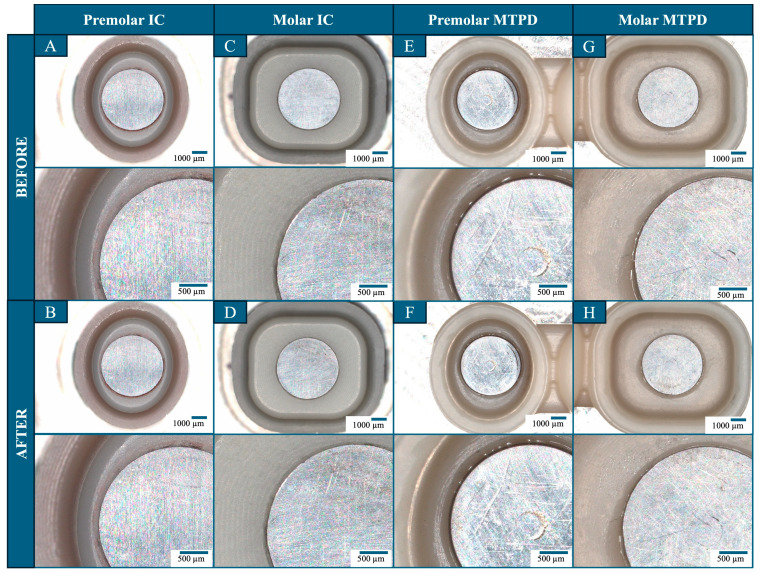
Surface evaluation of MA and KP fixed on the MTPD and ICs before and after the durability test. (**A**,**B**): Premolar IC; (**C**,**D**): molar IC; (**E**,**F**): MTPD premolar intaglio surface; (**G**,**H**): MTPD molar intaglio surface.

The surface evaluation results on the IC, OC part of the MTPD, MA, and KP microscopic images are presented in Figure 6. The upper images were at a smaller magnification covering all of the MA, KP, surrounding cement, and zirconia surface, whereas the lower images provide a representative close-up view. On the MA surface, slight irregularities were initially observed in all samples around the laser-welding area connecting the stainless-steel magnet bottom plate to the yoke. However, no significant changes were observed in the MA surface morphology before and after the durability tests. Similarly, the KP surface did not exhibit major changes due to wear on the surface. No deformation was observed in the cement areas fixing the MA–MTPD and KP–IC. Similarly, no obvious scratching or wear was observed on the MTPD or IC intaglio surfaces.

Based on the stability evaluation of the MTPD at 12 different single loading positions, no margin gap was detected when vertical loads were applied to the center and buccal part of the occlusal surfaces of both the premolar and molar OCs, before and after the durability test. There was no movement detected in all regions of interests when the load was applied to the pontic load-bearing areas. When a load was applied on the lingual load-bearing area of an abutment tooth’s OC, no movement was detected on the region of interest of the loading side, but slight micro-movements (≤67 µm) were detected on the opposite abutment-tooth side (Table 2). The recorded movement range did not demonstrate notable difference before and after receiving the durability tests. Overall, the movement was minimal, indicating stability of the MTPD structure.

## 4. Discussion

The fabrication of an MTPD utilizing the new UTMA system through a digital workflow can be successfully achieved with predictable results and favorable retentive properties. Based on the results of this study, the null hypothesis is accepted as no significant change was observed in the MTPD retentive force after the durability test.

Recent developments in magnetic attachment technology have provided solutions to previously reported limitations or drawbacks. A new magnetic attachment fabricated with stamping and high-frequency heat treatment technology successfully reduced the laser welding spots that were previously reported to be prone to deformation or leakage [3], reduced the fabrication cost, and involved a stainless-steel magnet bottom plate, which improved the retentive force [9]. However, the MA thickness of approximately 1.2 mm did not differ from that of previously available conventional cap-type magnetic attachments. Further development of the ring-type NdFeB magnetic circuit as well as the ring-shape stainless-steel magnet bottom plate made the fabrication of a UTMA with 0.6 mm MA thickness possible with the required performance. The UTMA system evaluated in this study is poised to become the thinnest prosthodontic attachment option, and its range of applications, including its use in MTPDs, can be increased.

Magnetic attachment thickness is a fundamental aspect to consider when planning prosthetic treatment design. Although they require a smaller occlusal clearance space than mechanical attachments [1,6], conventional-sized magnetic attachments still require a vertical space of at least 3 mm for the anterior region and 4 mm for the posterior region [1]. Such space requires extensive reduction in the denture and abutment tooth, resulting in the risk of denture and abutment-tooth fractures. The required space for UTMA, estimated by counting the sum of the MA (0.6 mm) and KP (0.4 mm), excels in this aspect and can resolve this limitation. A thin KP also offers the advantage of ease of removal, replacement, or renewal when necessary, as in the case of undergoing magnetic resonance imaging (MRI) scans, to avoid artifacts around the KP area [14,15]. Material size, shape, and position have been reported to affect the artifact area of MRI images [16]. Nevertheless, whether the MRI artifacts caused by the UTMA are smaller due to the minimized dimension must be clarified in future studies.

Conventionally, dental prostheses with magnetic attachments are fabricated using casting or direct cementation techniques [1,2,3]. Both techniques rely on the operator’s experience and skills, such as making wax patterns, casting, and KP cementation in the direct bonding method. The CAD/CAM technology used in the present study enables the fabrication of predictable and well-fitting dental prostheses [17]. Designing an MTPD intaglio surface for MA/KP housing or fixation space requires a combination of dental and general CAD software. To ensure the best fit, the cement space of the housing for UTMA components and the space between the ICs and OCs was determined by trial and error. As is demonstrated in Figure 4, fixation of the KP within the IC and the MA in the MTPD fabricated with CAD/CAM can be achieved, as proven by the MTPD–IC margin evaluation before and after MA and KP fixation. Restoration fit is also a factor contributing to stability [18].

The MTPD retentive force is approximately equal to the sum of the retentive forces of the fixed UTMA. In other words, the CAD/CAM fabrication process can provide the desired retention force for the MTPD. However, whether the retentive-force value of the MTPD is sufficient for clinical use remains unclear. The retentive-force value of the MTPD is likely to be satisfactory in clinical use, given the reports in which the acceptable retentive force of removable dental prostheses ranged from around 2.3 to 15.7 N [19,20,21,22]. With the aid of a rapidly growing digital workflow in dentistry, delivering an MTPD utilizing a UTMA might be carried out in a single visit using subtractive/additive-manufacturing-compatible materials. It would be beneficial for cases where a removable dental prosthesis is preferred for hygiene and easier maintenance purposes, especially when the presence of a denture base/plate extension is not preferred, and for elderly patients or those with limited dexterity.

The MTPD essentially requires that the OCs are not separated from the abutments during their functioning. This requirement is referred to as “stability”. The stability is attributed to the combination of supporting, bracing, and retention functions of the MTPD. Regarding such stability evaluation, the number of studies evaluating the stability or prosthetic movement of the telescopic crown system is scarce, and no standard mentions the exact range of movement that defines a removable denture as stable. Most in vitro studies reported retentive-force evaluations based on the aforementioned factors [22], whereas clinical studies usually reported treatment outcomes from patient-based subjective assessments [23,24,25]. In the present study, stability was evaluated by assessing the margin gaps following MTPD movements when a single-spot 100 N load was applied to a specific site on the occlusal table. When applying the load to the lingual side of the premolar and molar abutments, gaps ranging from 0 to 67 µm were observed on the crown opposite to the loading site, owing to torsional force. However, no gaps were detected at other loading and measuring sites. This is a typical characteristic of magnetic attachments, allowing for slight movement and re-positioning, thus reducing torsional/rotational stress on the abutment tooth. Clinically, the force applied during function would be more complex with more than one contact spot, and the mastication range of movement and opposing tooth anatomy would differ individually. Nakagawa et al. [26] reported that zirconia telescopic crowns with a 6.5 mm wall height, 4° taper, and 0–10 µm space displaced around 150 µm under a 100 N load. Thus, the gap observed in this study may be acceptable to patients.

The main concern of a UTMA-retained MTPD is durability because the magnet components are very thin. The MTPD durability test by applying insertion–removal cycles with a 100 N load 10,000 times was performed using the same protocol as highlighted in our previous study on conventional-sized magnetic attachments [9]. According to Schwindling et al. [27], the number of cycles represents 13–14 years of clinical time. This method has been widely used in previous studies evaluating the durability of magnetic attachments and telescopic or double crowns [27,28,29]. Following the durability test, no significant difference in retentive force was observed compared to the initial value. This result is in agreement with the findings of previous studies that have reported a stable retentive force of the magnetic attachment [30] following insertion–removal cycles and load tests [9,31,32]. Based on the surface evaluation results, no remarkable surface defects were identified on the KP, MA, IC, and MTPD intaglio surfaces or margins. This finding confirms the constant retentive-force results because surface deformation, especially at the MA–KP interface, can reduce the retentive force owing to gap formation [2,3]. Applying a UTMA to retain a telescopic/double crown might be beneficial compared to the conventional technique, as it does not rely on frictional forces of the IC and OC walls, which wear over time. Thus, when repair is necessary, it can be performed by removing the KP from the IC, instead of invasively removing/destroying the cemented IC from the abutment tooth.

The morphology of the UTMA evaluated in this study was without any retentive undercut or wing and was approximately 50% thinner than the thickness of the conventional MA. This design results in a small vertical bonding area with the prosthesis, which makes the attachment susceptible to shear stresses. Debonding of the MA from the prosthesis is also a concern for overdentures utilizing magnetic attachments [33]. However, no debonding was observed for the 10 MAs fixed using dual-cure resin cement in the five MTPDs in this study, demonstrating that an adequate fixation strength between the UTMA (stainless steel) and MTPD (zirconia) can be achieved through micromechanical (sandblasted surface) and chemical bonding (MDP-based primer and dual-cured resin cement). In this study, zirconia was utilized due to its suitability for CAD/CAM processes and has been widely adopted in recent studies on telescopic and double crown systems due to its superior wear resistance, fracture toughness, good esthetics, and less bacterial accumulation [22]. The appropriate selection of adhesive protocols with respect to selected materials for ICs and OCs should be performed meticulously to achieve prosthesis longevity.

Nevertheless, this study has some limitations. The outer morphology of the MTPD was simplified to facilitate in vitro evaluations, whereas in the clinical condition, the prosthesis morphology corresponds to tooth anatomy adjusted for opposing tooth contacts. Therefore, the stress distributions affecting the UTMA and MTPD structures may differ. Moreover, the load applied for MTPD stability was limited to the occlusal direction, whereas clinically, complex jaw movements and occlusal surface morphology result in multidirectional loading. Other environmental factors that should be considered are the presence of saliva, an acquired pellicle, and thermal changes in the oral cavity, which may influence the retentive, stability, and durability properties. The sample size evaluated in this study was relatively small, and further studies with formal statistical power calculations are warranted. Clinical research on a UTMA-retained MTPD comprising its clinical outcomes and crossover with other prosthesis types is recommended to justify its performance after further in vitro evaluation.

In addition, the retentive force of a UTMA ranged from 3.02 to 3.61 N, with a magnetic field strength of 127.77–181.07 mT. Within the 10 UTMAs evaluated, both ranges still have wide variations. A higher magnetic field strength typically represents a higher attractive force; however, the values obtained in this study did not align with that concept. Further quality control of the MA fabrication process, including ensuring a complete contact interface between the KP and the MA and optimization of the magnetic circuit formation, is also required. In silico studies such as 2D and 3D visualization using finite element method of the magnetic circuit should be considered to further understanding in this area, including understanding of its characteristics.

## 5. Conclusions

Based on the results of this study, the following can be concluded:

- Contemporary MTPDs utilizing UTMAs fabricated using a digital workflow exhibit favorable properties.
- The novel UTMA system possesses an adequate retentive force despite its thin structure and provides a broad opportunity for utilization in prosthodontic treatment.
- The assembly might be considered to be a feasible dental prosthesis due to its constant retentive force, adequate stability under loaded conditions, and durable properties.

## Figures and Tables

**Table 1:** Basic properties of UTMA used and retentive force of MTPD. The bottom row with bold numbers represents the mean ± standard deviation of the values displayed in the columns above.

UTMA		MTPD Retentive Force (N)		
Magnetic Field Strength (mT)	Retentive Force (N)	Sample No.	Baseline (Mean ± SD)	After Durability Test (Mean ± SD)
156.00	3.12	1	6.78 ± 0.24	5.97 ± 0.05
158.83	3.02			
181.07	3.21	2	7.26 ± 0.12	7.1 ± 0.02
153.87	3.32			
163.07	3.04	3	5.82 ± 0.03	5.66 ± 0.02
174.22	3.62			
133.50	3.41	4	7.05 ± 0.02	7.44 ± 0.01
138.40	3.31			
160.50	3.22	5	7.41 ± 0.07	7.12 ± 0.05
127.77	3.21			
154.72 ± 17.13	3.25 ± 0.18		6.86 ± 0.63	6.66 ± 0.79

**Table 2:** Stability of MTPD: gaps observed from the buccal side under loaded conditions (L: lingual; C: center; B: buccal).

Load-Bearing Area		Before/After Durability Test	Measured Locations (Min–Max Values in µm)					
			a	b	c	d	e	f
Premolar	L	before	0	0	0	19–29	32–55	25–52
		after	0	0	0	18–41	33–63	26–45
	C	before	0	0	0	0	0	0
		after	0	0	0	0	0	0
	B	before	0	0	0	0	0	0
		after	0	0	0	0	0	0
Molar	L	before	0–67	0–53	0–36	0	0	0
		after	0–63	0–53	0–25	0	0	0
	C	before	0	0	0	0	0	0
		after	0	0	0	0	0	0
	B	before	0	0	0	0	0	0
		after	0	0	0	0	0	0

## Data Availability

The original contributions presented in this study are included in the article. Further inquiries can be directed to the corresponding author.

## Abbreviations

The following abbreviations are used in this manuscript:

MA	Magnet assembly
KP	Keeper
UTMA	Ultra-thin magnetic attachment
MTPD	Magnet-retained telescopic partial denture
IC	Inner crown
OC	Outer crown
